# A Modified Nickel Titanium Space Regainer Using an Open Coil Spring in a Pediatric Patient: A Case Report

**DOI:** 10.7759/cureus.72395

**Published:** 2024-10-25

**Authors:** Sushmita Shan, Kavitha Swaminathan, Vivek K, SelvaKumar Haridoss, Kritiga Kumar

**Affiliations:** 1 Pediatric and Preventive Dentistry, Sri Ramachandra Dental College and Hospital, Sri Ramachandra Institute of Higher Education and Research, Chennai, IND

**Keywords:** congenital hypodontia, early intervention, light continuous force, modified orthodontic technique, niti open coil spring, pediatric orthodontics, posterior crossbite, primary molar extraction, space maintainers, space regaining

## Abstract

This case report introduces a novel modification in the space-regaining process using a nickel-titanium (NiTi) open coil spring in a five-year-old male patient with early primary molar loss. The space deficiency, which led to occlusal discrepancies such as posterior crossbite, was efficiently managed using the NiTi wire and open coil spring, which is known for its superelasticity. The modification involved the use of a NiTi open coil spring, enabling effective space regaining and stabilization with band and loop space maintainers. This technique minimized patient discomfort and reduced treatment time. The report highlights the advantages of this modified approach in pediatric interceptive orthodontics, particularly in addressing space loss early and effectively.

## Introduction

The premature loss of primary teeth presents a complex challenge with significant implications for the dental arch and the alignment of permanent teeth due to space loss [1]. The loss of primary teeth causes adjacent permanent teeth to drift into the space. This unplanned movement can disrupt the eruption path of permanent successors, potentially resulting in malocclusion, misalignment, and increased orthodontic complexity [1]. Space regaining is critical to ensure the proper eruption and functional occlusion of permanent successors [2]. Traditional methods involve the use of fixed or removable appliances that often require significant patient compliance and long treatment times [3]. However, recent advancements in orthodontic techniques offer innovative alternatives to conventional space maintenance methods [3]. Nickel-titanium (NiTi) open coil springs, which apply light and continuous forces, provide a promising option for space regaining, especially in younger patients [3]. This alloy, known for its superelasticity and shape memory properties, offers several advantages over conventional appliances [4]. The light, continuous forces generated by NiTi springs are not only effective in maintaining and regaining space but also contribute to patient comfort by minimizing the risk of sudden, discomforting pressure [4]. The exploration of NiTi open coil springs represents a potential paradigm shift in space maintenance, with implications for improving treatment outcomes and reducing the burden of patient compliance in orthodontic practice. In this case, we present a modified approach to space regaining using a NiTi open coil spring, offering a more efficient and less invasive method for addressing space loss. This modification was designed to enhance treatment efficiency by reducing the number of required adjustments and improving patient comfort.

## Case presentation

**Figure 1:**
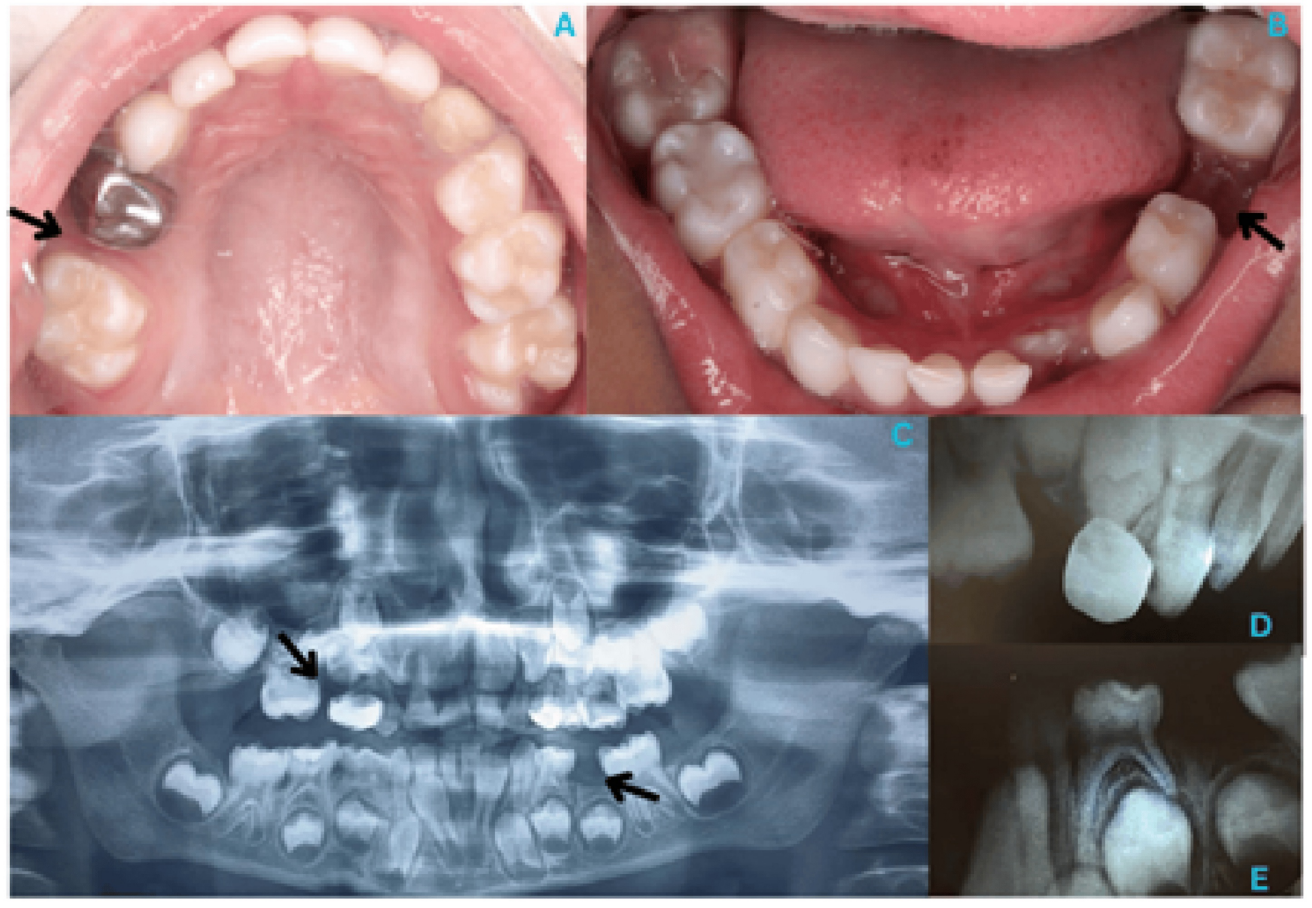
Preoperative Photograph and Radiographs (A) Preoperative maxillary occlusal view. (B) Preoperative mandibular occlusal view. (C) Orthopantomogram - Huckaba analysis revealed a space loss of 3 mm in the region of tooth 55 and 0.7 mm in the region of tooth 75. (D) Intraoral periapical radiograph in relation to tooth 55. (E) Intraoral periapical radiograph in relation to tooth 75.

A five-year-old male presented to the Outpatient Department of Pediatric & Preventive Dentistry with a chief complaint of missing right upper and left lower back teeth. The patient had a history of adenotonsillar hypertrophy and exhibited a mouth-breathing habit, for which he was undergoing pharmacological management. His dental history included a laser lingual frenectomy performed two years ago and a tooth extraction ten months prior. Clinical examination revealed the absence of the upper right primary molar (tooth 55) and lower left primary molar (tooth 75), with mesial tilting of the adjacent permanent teeth and a unilateral posterior crossbite on the right side (Figure 1).

Radiographic evaluation confirmed space loss and revealed the congenital absence of the mandibular central incisors (teeth 31, 41). Intraoral and radiographic examinations (Figure 1) with Huckaba analysis indicated a space deficiency of 0.7 mm at tooth 75 and 3 mm at tooth 55, along with mesial tilting of the upper right first permanent molar (tooth 16) and the lower left first permanent molar (tooth 36). The space loss was due to the early extraction of primary molars, compounded by a posterior crossbite and the congenital absence of the permanent mandibular central incisors.

**Figure 2:**
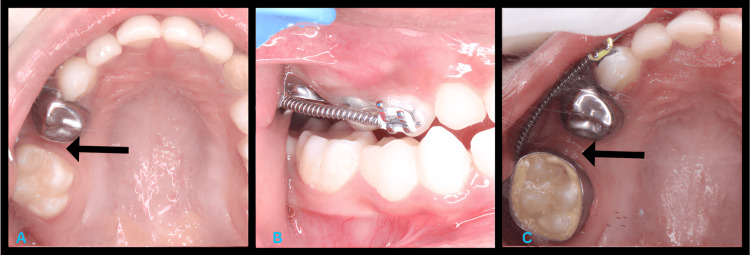
The Use of a NiTi Wire and an Open Coil Spring as a Space Regainer (A) Preoperative maxillary occlusal view. (B) Intraoperative right lateral view. (C) Postoperative maxillary occlusal view.

The primary treatment goals were to regain space using a modified approach with NiTi open coil springs and to maintain the space with band and loop maintainers. The treatment plan was explained to the patient's parent, and informed consent was obtained. The modification involved using NiTi wire and open coil springs in a way that minimized patient discomfort and facilitated faster space regaining. A NiTi wire and an open coil spring were placed between the upper right first permanent molar (tooth 16) and the upper right primary canine (tooth 53) using an MBT bracket (Figure 2). Since the upper right primary first molar (tooth 54) had a stainless steel crown, the bracket could not be bonded to it; therefore, the bracket was placed on the upper right primary canine (tooth 53).

**Figure 3:**
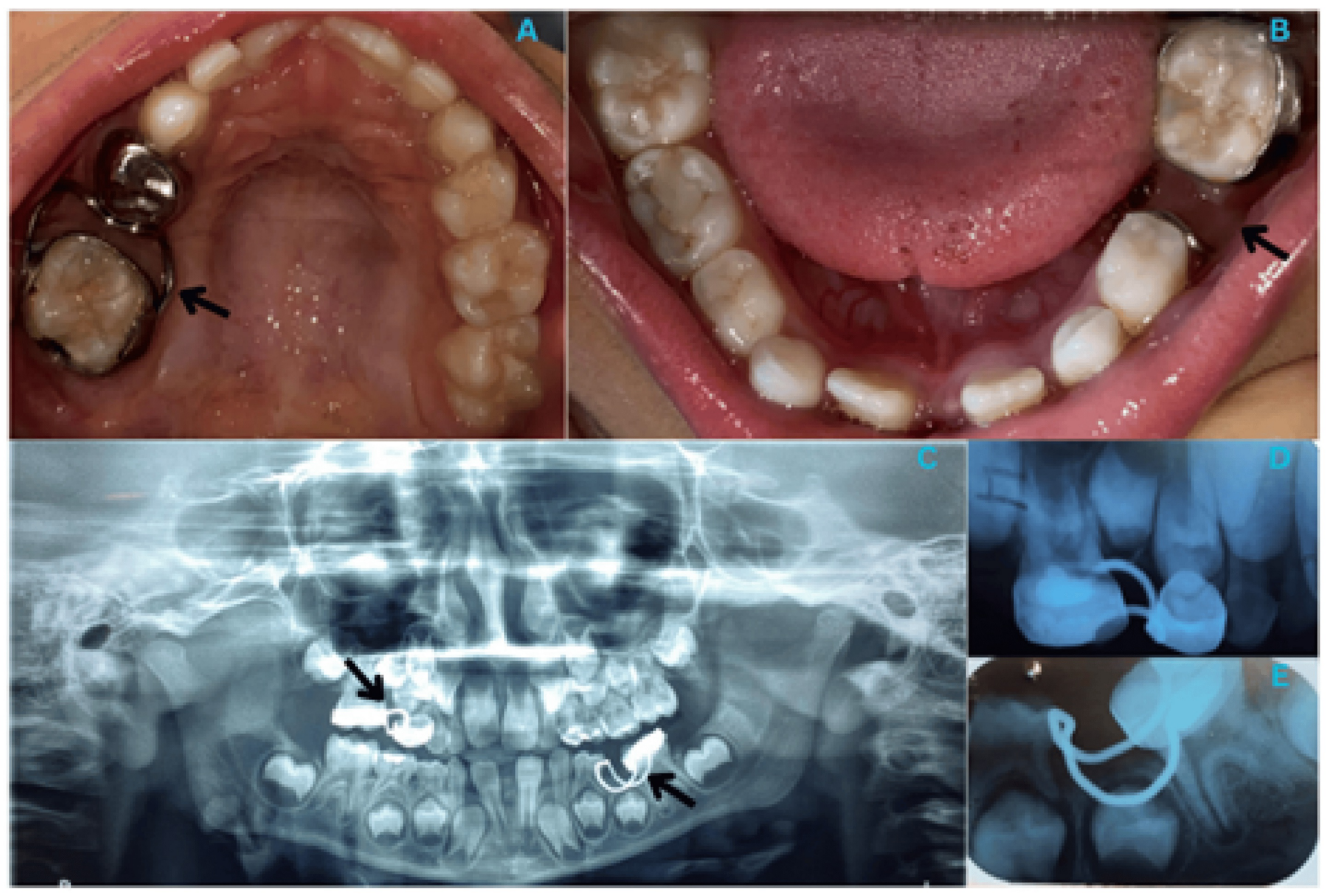
Postoperative Photograph and Radiographs (A) Postoperative maxillary occlusal view. (B) Postoperative mandibular occlusal view. (C) Orthopantomogram. (D) Intraoral periapical radiograph in relation to tooth 55. (E) Intraoral periapical radiograph in relation to tooth 75.

The spring was activated with a 0.014 NiTi wire, applying continuous force to regain the lost space. This modification involved extending the coil spring by 2 mm to ensure a larger activation range, reducing the need for frequent adjustments. The patient experienced mild pain and discomfort, for which an analgesic was prescribed. Over two weeks, 2 mm of space was regained, with a total of 6 mm eventually achieved in the region of the upper right primary molar. A band with a buccal tube was fitted on the upper right first permanent molar (tooth 16) to control mesial tilting. Following this, a band and loop space maintainer was secured in the area of tooth 55 to maintain the regained space. Another band and loop space maintainer was placed in the area of tooth 75 (Figure 3).

The patient is under regular follow-up to monitor progress, ensuring proper eruption and alignment of the successor teeth. The patient is also being monitored to address the mouth-breathing habit, which is due to Grade I adenotonsillar hypertrophy. The modified approach significantly shortened the treatment time while maintaining patient comfort. This method ensured that the space was maintained without the need for further complex interventions.

## Discussion

The modification in this case report focused on the strategic use of NiTi open coil springs to regain space more efficiently and comfortably. Traditional space regainers, such as removable appliances or more complex orthodontic devices, often require frequent adjustments and extended treatment times [1,3]. However, by using NiTi open coil springs with extended activation lengths, this case achieved faster space regaining with fewer patient visits and adjustments [2]. This approach leverages the unique properties of NiTi alloy, which are essential in optimizing both the efficiency and comfort of orthodontic treatments. NiTi open coil springs are known for their superelasticity and shape memory characteristics, enabling them to exert light, continuous forces over a prolonged range of activation [4]. This property is particularly advantageous in space regaining, as it eliminates the need for frequent adjustments typically required by traditional space maintainers, streamlining the treatment process and enhancing patient comfort [5,6]. The superelastic nature of NiTi springs allows them to deliver a gentle, continuous force that is well-tolerated by pediatric patients, who may have lower tolerance for traditional appliances [7,8]. Additionally, the use of band and loop space maintainers to stabilize the regained space highlights the importance of maintaining space until the eruption of permanent teeth, preventing further occlusal disturbances and ensuring proper alignment [9,10].

This case reinforces the value of early intervention in pediatric orthodontics, as untreated space loss can lead to more complex malocclusions requiring extensive treatment later in life. The application of NiTi open coil springs represents a significant advancement in managing space loss due to premature primary tooth loss, offering a more efficient and patient-friendly alternative to traditional methods. This modified approach not only improves treatment outcomes and patient comfort but also emphasizes the need for early intervention and comprehensive space management strategies. Early identification of space loss, combined with efficient and patient-friendly techniques like the modified NiTi open coil spring method, can prevent the need for future orthodontic corrections [11]. Future research and clinical trials will be essential to further validate these findings and refine treatment protocols, enhancing the standard of care in orthodontics.

## Conclusions

The modification of space regaining using NiTi open coil springs in this case provided an effective, patient-friendly solution for managing space loss in a pediatric patient. The reduced need for frequent adjustments, combined with the continuous, light force applied by the springs, allowed for faster space regaining and improved overall treatment efficiency. This approach serves as a valuable modification for similar cases, offering a simple yet effective method for addressing space deficiencies and occlusal issues in pediatric patients.
